# A Spatial Regression Analysis on the Effect of Neighborhood-Level Trust on Cooperative Behaviors: Comparison With a Multilevel Regression Analysis

**DOI:** 10.3389/fpsyg.2019.02799

**Published:** 2019-12-19

**Authors:** Daisuke Takagi, Takahito Shimada

**Affiliations:** ^1^School of Public Health, Graduate School of Medicine, The University of Tokyo, Tokyo, Japan; ^2^National Research Institute of Police Science, Kashiwa, Japan

**Keywords:** trust, cooperative behavior, spatial Durbin model, spatial regression analysis, multilevel model

## Abstract

There is no reason to suppose that neighborhood effects based on residents’ trust vary according to administrative boundaries. We examined the relationship between neighborhood trust and cooperative behaviors using the spatial Durbin model which assumed that people are influenced by closer neighbors regardless of administrative boundaries, comparing the results with those of the multilevel model. We used data from 476 residents in Arakawa Ward, Tokyo, Japan. For each respondent, we assigned a unique ‘neighborhood trust’ value weighted by the inverse distance between the respondent and all other respondents as an independent variable. The dependent variables were perceived neighbors’ cooperative behaviors and respondents’ own cooperative behaviors. The spatial Durbin model showed that spatially weighted neighborhood trust was positively associated with cooperative behaviors. Meanwhile, the multilevel models did not show the statistically significant effect of neighborhood trust. We concluded that the spatial model might model the neighborhood effects in society more precisely.

## Introduction

In the fields of neighborhood research such as social psychology, public health, and criminology, researchers have studied the effects of ‘place’ as the source of social/environmental influences on people’s behaviors, well-being, and quality of life. For example, the previous studies have demonstrated that the amount of social capital/cohesion (Sampson et al., 2002; Kawachi et al., 2013), economic situation (Robert, 1999; Ross, 2000), welfare policy (Gnanasekaran et al., 2008), and quality of social milieu (Jia et al., 2009) vary among states or municipalities and that they explain a certain level of variance in the health and safety of residents in different areas. In these studies, geographical boundaries such as states, counties, municipalities, school districts, and police districts are used to define the sources of neighborhood effects on residents (Sampson et al., 2002; Takagi, 2013). Given that social policies differ among administrative districts, it is natural that the effects of welfare policy and social milieu on people’s health and safety vary depending on such ministerially defined boundaries.

However, determining neighborhood psychosocial effects such as social capital and cohesion according to administrative boundaries is arbitrary. While neighborhood social capital and cohesion affect residents’ health and safety through social interaction, social support, collective efficacy, informal social control, and cooperative behavior among neighbors (Sampson et al., 1997; Berkman et al., 2014), there is no reason to suppose that these differ according to administrative boundaries (Morenoff et al., 2001). The purpose of this paper is to propose an analytic framework using a spatial definition of ‘neighborhood’ defined by physical distances among residents instead of by administrative boundaries when analyzing social influences. Through this, we sought to bring the perspective of spatial analysis to neighborhood research in the psychology field.

Previous studies have shown that ‘neighborhood-level trust’ is associated with residents’ health and safety. For example, Kawachi et al. (1999) combined multiple datasets and examined the correlation between the percentages of people who did not trust others and those with low self-rated health using data aggregated by states. In their analysis, they found that the proportion of people who were mistrustful was positively associated with those who had poor self-rated health (*r* = 0.71). Snelgrove et al. (2009) used 250 postcode areas in the United Kingdom as neighborhood units and examined the relationship between trust aggregated at neighborhood-level and individual-level self-rated health using a multilevel model. They found a contextual effect wherein those who live in a neighborhood with a high level of trust were likely to report good self-rated health. Mazerolle et al. (2010) divided the entire city of Brisbane, Australia, into 82 statistical local areas (SLA) and examined the association of SLA-level collective efficacy consisting of social cohesion and trust with self-reported violent victimization. Their results showed that individuals living in areas with high collective efficacy were statistically significantly less likely to be victims of violent crime. Roh and Lee (2013) used a multilevel model to investigate the association between the aggregate-level generalized trust and individual-level robbery victimization using data from 56,071 individuals residing in 56 countries. They demonstrated that country-level generalized trust was negatively related to individual-level robbery victimization, after adjusting for individual-level covariates such as income and lifestyle.

In communities with a high level of neighborhood trust, mutual support and cooperative behaviors among neighbors are likely to increase, resulting in positive effects such as health promotion and crime control. Trust is a facet of one’s expectations of others’ intentions (Yamagishi, 2011). The belief that other people will behave as expected is based on their competence or intentions (Barber, 1983). Yamagishi (2011) and the present study take the view that trust is one’s expectations about whether others have intentions to exploit him/her or act selfishly, not competence to do so. According to Gambetta (1988, p. 217), when we trust someone in this sense, “we implicitly mean that the probability that he will perform an action that is beneficial or at least not detrimental to us is high enough for us to consider engaging in some form of cooperation with him.” A person who trusts that others will cooperate may believe that he/she will not be exploited by them when cooperating; therefore, he/she can cooperate with others (Ahn and Ostrom, 2008). Thus, in neighborhoods where most people trust each other, it is expected that residents will tend to cooperate with each other, and that a person living in such a neighborhood perceives a high rate of cooperative behaviors among neighbors.

In addition, people who are trusted by others are more likely to cooperate on reciprocity with the trustors. Many trust game studies have confirmed that when the trustor shows a trusting behavior, the trustee generally tends to reciprocate to it (Berg et al., 1995; Pillutla et al., 2003; Eilam and Suleiman, 2004). Therefore, it is expected that people surrounded by neighborhood residents demonstrating high trust are more likely to cooperate with their neighbors. Thus, as a social consequence of residents living in a neighborhood where neighboring residents’ trust is high, both perceived surrounding neighbors’ cooperative behaviors and their own cooperative behaviors are expected to increase.

As mentioned previously, while a multilevel model using aggregated data at municipal level would be appropriate when examining the effect of a place characteristic such as a social policy, a model incorporating spatial proximity among residents may be useful for capturing the effects of ‘grass-roots’ social interactions. The relationship between neighborhood trust and cooperative behaviors is an appropriate candidate for the spatial proximity theory; it is reasonable to suggest that trust of those living spatially close by–not just of those living in the same neighborhood–is more likely to be related to one’s own cooperative behaviors and perception of neighbors’ cooperation.

In this context, who are the neighborhood ‘others’ in neighborhood studies? In other words, what is the geographical range that defines a neighborhood? Previous studies examining the relationships between neighborhood social capital/cohesion and health/crime victimization outcomes using multilevel models have incorporated a variety of spatial scales: countries (Roh and Lee, 2013), states (Desai et al., 2005), census areas (Blakely et al., 2006), municipalities (Islam et al., 2008), school districts (Takagi et al., 2013), and zip code areas (Wen et al., 2005). However, there are two problems in defining a neighborhood by geographical boundaries. First, if the size (geographical definition) of the neighborhoods differs, the data may lead to different analysis results depending on that size. This problem is known in geography as the modifiable areal unit problem (MAUP) (Fotheringham and Wong, 1991; Cockings and Martin, 2005). An example is when Mobley et al. (2008) investigated the neighborhood-level factors associated with mammographies among women in California using multilevel models. They conducted their modeling with four different geographical units: postal zip code areas (area-level *n* = 1450), primary care service area (*n* = 333), the medical service study area (*n* = 519), and county (*n* = 57). The results showed that the associations of neighborhood-level racial segregation index and the poverty rate with the individual-level outcome (i.e., mammography examination) varied greatly depending on the areal units used for the analyses. Tarkiainen et al. (2010) conducted two multilevel model analyses for the relationship between neighborhood-level independent variables, such as the proportion of manual workers, and individual-level mortality, using two different areal units in Helsinki (70 Districts and 258 smaller sub-districts). They reported that the effects of neighborhood-level variables on mortality was slightly greater in the model with a smaller areal unit.

Although, as mentioned above, some studies using the multilevel model framework empirically examined the changeability of results due to the definition of neighborhood, as long as certain geographical boundaries are used, the problem of ignoring the spatial proximity between residents occurs regardless of the geographical unit. As Takagi et al. (2012) stated, people living near the border of an administrative boundary are more affected by their neighbors living in close by but in the next district than they are by residents distant to them but in the same district. Thus, dividing a neighborhood with an administrative boundary ignores the social influences of proximity, and it arbitrarily posits the premise that people are influenced by others who live in the same district. This problem can be briefly expressed in the following statement by Morenoff et al. (2001, p. 522): “Two families living across the street from one another may be arbitrarily assigned to live in different “neighborhoods” even though they share social ties.” Defining a neighborhood based on administrative boundaries ignores grass-root level social interactions and the spatial spillover effect among residents across neighborhood boundaries.

Takagi (2013) defined neighborhood not by administrative boundaries but by the distance between each participant in their survey. They defined ‘neighborhood participants’ by some geographical ranges (e.g., 50 meters, 100 meters, 500 meters, etc.) from each participant and examined the relationship between neighbors’ trust and likelihood of victimization in a crime. Chaix et al. (2005) defined each participant’s nearest 100, 200, 500, 1,000, and 1,500 other participants as his/her ‘neighbors’ and investigated the association between neighbors’ income and his/her mental health. Both studies showed that neighborhood effects were maximized when using the smallest definition of neighborhood. However, in the field of psychology, there are no neighborhood studies that define neighborhood not by administrative boundaries but by distance. In addition, although the previous studies defined multiple neighborhoods regardless of administrative boundaries by using various geographical ranges (Takagi, 2013) and number of surrounding participants (Chaix et al., 2005), and compared neighborhood effects among them, these methods assumed zero influence from people outside of the defined neighborhood, as in the multilevel model framework using administrative boundaries.

To address the above-mentioned limitations in neighborhood effect studies, we defined the neighborhood for each resident using inverse distances to other residents rather than defining it by geographical boundaries. It used the simple idea that regardless of boundaries people are strongly influenced by those nearer to them and less influenced by those more distant. This definition of neighborhood by the inverse distances among respondents is in line with Tobler’s first law of geography: “everything is related to everything else, but near things are more related than distant things” (Tobler, 1970, p. 236). To examine this, we plotted the respondents to the postal survey on a geographic information system (GIS) and created a spatially weighted matrix to represent the inverse distances among all the respondents. Following this, we used the spatial Durbin model (Anselin, 1988) to examine the relationships between neighborhood trust and perceived neighbors’ cooperative behaviors/respondent’s own cooperative behaviors. Thereafter, we compared the results of the spatial analyses with those of multilevel models.

## Materials and Methods

### Data

This study used data from the second wave of a panel survey (Survey on Neighborhood Crime Prevention and Environmental Problems) conducted in Arakawa Ward, Tokyo. Arakawa Ward is a metropolitan area with a population of 203,296, area of 10.20 km^2^, and population density of 19,931.0/km^2^ (2010 Census). In 2009, the researchers conducted a postal survey of 1,000 men and women aged 20 to 69 years (the first-wave survey). In this first-wave survey, the researchers used two-stage random sampling of eligible voters. In the first stage, 10 voter registration ledgers from 32 ledgers were randomly sampled. In the second stage, 100 individuals from each voter registration ledger (100 × 10 = 1,000) were randomly sampled. The response rate was 48.0% (*n* = 480). In 2011, a postal survey was sent to 469 traceable respondents who responded to the first-wave survey and 500 new men and women aged 20 to 69 years (the second-wave survey). For the 500 new targets in the second-wave survey, the researchers randomly sampled 50 individuals from each voter registration ledger chosen in the first wave survey (50 × 10 = 500). As a result, a questionnaire was sent to 969 (469 + 500) individuals in the second wave survey with the response rate of 57.6% (*n* = 558).

**FIGURE 1:**
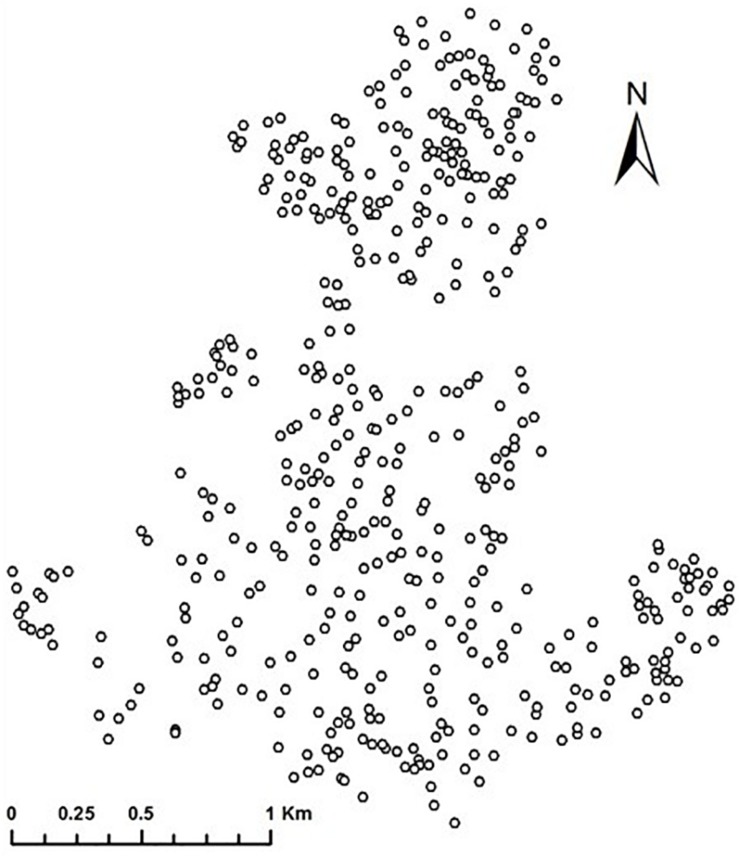
Spatial distribution of respondents.

In the analyses, we omitted 33 respondents with missing values on variables used. Also, for respondents who shared the same address because they lived in the same apartment building, the distance between them equaled zero and the spatial-weighting matrix using the inverse-distance between respondents could not be created. Therefore, we randomly chose one respondent from among those at the same address. As a result, we excluded 49 respondents from the analyses. Finally, we used data from 476 respondents for the analyses. Figure 1 represents the spatial distribution of respondents used in the analyses.

In the multilevel model analyses conducted for later comparison, 476 respondents resided in 26 neighborhoods (18.3 respondents on average per neighborhood, standard deviation was 7.5). The neighborhood unit of the multilevel models in this study was a *choumoku*, which is the smallest ministerial defined areal unit in Japan. The average size of a *choumoku* in Arakawa Ward was 0.1962 km^2^.

### Measurements

#### Dependent Variables

Since the original purpose of the survey was to examine the associations between neighborhood social relationships and crime victimization, measurements of cooperative behaviors included items related to crime prevention in the neighborhood. For perceived neighbors’ cooperative behaviors, the survey asked respondents about their perception of neighborhood watch, which is one of the most common informal social controls conducted in the neighborhood in Japan (Richey, 2005): “Patrol by residents on foot or bicycle” and “Residents watching children making their way to and from school.” Participants responded on a four-point Likert scale (1 = not seen at all, 2 = not seen very much, 3 = sometimes seen, 4 = frequently seen). In the analyses, we used the average of these two items (Cronbach’s alpha = 0.613). Since neighborhood watches can be conducted without governmental approval or financial support (Garofalo and McLeod, 1989) and are based on collective action among residents (Richey, 2005), it is appropriate to set neighbors’ participation in such activities as a dependent variable for the purpose of examining the relationship between trust and cooperative behaviors in the neighborhood.

A previous study measured the neighborhood watch variable as a dichotomous variable, representing whether neighbors joined with other neighbors to prevent crime (Richey, 2005). Other studies asked respondents whether “neighborhood or community watches” and “volunteer surveillance of residential neighborhoods by residents” would deter crime. This was done to measure the perceived impact of informal control on deterrence of committing crime and demonstrate their reasonable internal consistency (α = 0.73) and high factor loadings on a factor in factor analysis (0.81 for both items) (Jiang et al., 2010, 2012). However, the respondents’ perceptions of effectiveness of neighborhood watches are inappropriate for measuring perceptions of how much such cooperative behaviors are conducted in the neighborhood. While some previous studies dealt with neighborhood watches, there are no existing measurements that fit the purpose of the present study; therefore, this study created the above two specific items. These items reflect the fact that neighborhood watches in Japan are carried out by volunteers traveling by foot or bicycle or when children are walking to and from school (National Police Agency, 2014), enabling respondents to recall more specific situations than the above previous studies.

In addition, respondents’ cooperative behaviors was measured by asking the following items: “Participation in a self-governing association and discussion of various facets of the neighborhood,” “When seeing a suspicious person in neighborhood, questioning him/her,” “Usually, I care when unusual things (strange sounds or suspicious figures) can be seen around neighbors’ houses,” “Cooperating with neighbors to clean up parks and roads in the neighborhood,” and “When seeing a car or bike that is illegally parked in the neighborhood, notify the police.” Participants again responded on a four-point Likert scale (1 = never, 2 = not so much, 3 = sometimes, 4 = often). In the analyses, we used the average of these five items (Cronbach’s alpha = 0.786).

In questions about values and attitudes, Japanese respondents are more likely than respondents from Taiwan, Canada, and the United States to choose the midpoint on scales (Chen et al., 1995). Therefore, for the Likert scale items, this study adopted an even number of options without a midpoint. Although six possible responses is the usual practice for an even-numbered Likert scale (DeVellis, 2012), this study used 4-point scales in order to reduce respondents’ response time and cognitive load.

#### Independent Variable

The present study measured respondents’ trust in others using the following single item extracted from Yamagishi and Yamagishi’s (1994) General Trust Scale: “Most people are trustworthy.” This item indicated the highest or second highest factor loading in the factor analyses of the General Trust Scale items (Yamagishi and Yamagishi, 1994; Lazzarini et al., 2008; Yamagishi et al., 2015). Participants responded on a four-point Likert scale (1 = strongly disagree, 2 = disagree, 3 = agree, 4 = strongly agree). This was used as a continuous variable in the analyses.

Previous studies have demonstrated that this single item of trust was statistically significantly associated with lifestyle (Xue and Cheng, 2017), depressive symptoms (Qin and Hsieh, 2018), and happiness (Yamada et al., 2013). In addition, a Turkish study using this item showed that people’s trust pronouncedly decreased after the failed coup d’état attempt, as theoretically expected (Akkemik et al., 2019). These studies suggest the criterion-related validity of this item.

#### Covariates

We adjusted the analyses for sociodemographic covariates including the sex of the respondents, educational level, perceived social class, residence year, and the presence of primary school children in the household (as this has been reported associated with crime prevention participation in local communities) (Hope and Lab, 2001; Lee and Cho, 2018). In particular, socioeconomic positions indicators such as educational level and perceived social class can also be confounding factors related to both dependent and independent variables (e.g., selection of residential area). While age has also been reported to be associated with crime prevention-related cooperative behaviors (Hope and Lab, 2001; Lee and Cho, 2018), it was not included in the analyses in order to avoid multicollinearity with the variable of residence year. For educational level, we coded respondents with college/vocational school or less education as 0 and those with a university degree or higher as 1. Since asking about actual income tends to lower response rate, we asked respondents about their perceived social class as an alternative indicator of their economic situation: “If Japanese society is divided into five groups, to which group do you think you belong?” We obtained responses from four predetermined categories (1 = lowest, 2 = lower middle, 3 = upper middle, 4 = highest). We considered residence year as a continuous variable. For the presence of primary school children in the household, we coded respondents who lived with primary school children as 1, without as 0.

As mentioned previously, the dependent variables of the survey included cooperative behaviors relevant to crime prevention. Therefore, we adjusted for fear of crime as a potential confounder of the independent variable (i.e., trust) and the dependent variables (i.e., cooperative behaviors) (Skogan and Lurigio, 1992). The fear of crime was assessed by asking about each respondent’s fears of “burglary,” “car or motorbike theft/car break-ins,” “bicycle theft,” “vandalism to cars or houses,” “street crime (purse-snatching, indecency, or extortion),” and “life-threatening crimes.” Participants responded on a four-point Likert scale (1 = do not feel anxious at all, 2 = do not feel anxious, 3 = feel anxious, 4 = feel highly anxious). In the analyses, the average of these six items was used (Cronbach’s alpha = 0.845).

### Statistical Analysis

We used the spatial Durbin model (Anselin, 1988) to examine the relationships between neighborhood trust and cooperative behaviors:

$$y=\rho \,\mathrm{Wy}+\beta_{1}\,X_{1}+\beta_{2}\,{\mathrm{WX}}_{1}+\beta_{3}\,X_{2}+\varepsilon$$

$$\varepsilon ∼N\,\left(0,\sigma^{2}\,I\right)$$

where *W* is the inverse-distance spatial-weighting matrix. ρ is the spatial autoregression parameter, representing the spatial dependency of neighbors’ dependent variable. β_1_ is the regression coefficient of the independent variable *X*_1_ (e.g., trust), and β_2_ is the regression coefficient of *other respondents’* independent variable *X*_1_ weighted by the inverse-distance weighting matrix. β_3_ is the regression coefficient of a covariate *X*_2_ (e.g., sex). ε is the error term having a mean of 0 and a variance of σ^2^.

The present study created the spatial-weighting matrix using inverse distances among respondents. That is, since there were 476 respondents in this study, we created a matrix of 476 × 476. Table 1 shows the dimension of the inverse-distance spatial-weighting matrix and the minimum and maximum of the inverse distance among respondents. For respondents living in the same apartment building, we randomly used data from one of them for the analyses and did not include respondents with 0 distance in the matrix.

**TABLE 1:** Summary of the inverse-distance spatial-weighting matrix.

Dimensions	476 × 476
Inverse distance	
Min	0.3144
Max	76.0237

Table 1 shows that the distance between the nearest respondents was 0.013154 km (1/76.0237) and that of the most distant respondents was 3.180662 km (1/0.3144). That is, in this matrix, large values are stored in dyads with a short distance, and small ones are stored in dyads with a long distance. In our analysis, we used the minmax-normalized matrix in which each element was divided by the minimum of the largest row sum and column sum of the matrix (Drukker et al., 2013).

For each respondent, we weighted other respondents’ values of trust according to the inverse distances among them. Following this, we assigned the average of the weighted values of all other respondents’ trust to each respondent as an independent variable. Thus, each respondent was assigned a unique exposure to neighborhood trust in which the inverse distances regardless of geographical boundaries defined neighborhood influence. In addition to trust, for each respondent, we also weighted other respondents’ dependent variable (i.e., perceived neighborhood residents’ cooperative behavior/respondent’s behaviors) by the inverse-distance spatial-weighting matrix and included them in our statistical models as the spatial autoregression term.

Additionally, multilevel models were conducted for comparison. In these models, individuals were treated as level-1 and neighborhoods were level-2. The unit of the neighborhood was the *choumoku*. The level-1 model was represented by the following equation that predicts the dependent variable of individual *i* within neighborhood *j* (Raudenbush and Bryk, 2002):

$$y_{\mathrm{ij}}=\beta_{0\,j}+\beta_{1\,j}\,X_{1\,\mathrm{ij}}+\beta_{2\,j}\,X_{2\,\mathrm{ij}}+r_{\mathrm{ij}}$$

where β_0__*j*_ is the intercept, β_1__*j*_ is the regression coefficient of the individual-level explanatory variable *X*_1__*ij*_ (e.g., trust), β_2__*j*_ is the regression coefficient of an individual-level covariate *X*_2__*ij*_ (e.g., sex), and *r*_*ij*_ is the level-1 residuals. The intercept β_0__*j*_ is differed for each neighborhood, that is, representing the variation in the average value of the dependent variable among neighborhoods. The level-2 model that predicts β_0__*j*_ using the neighborhood-level explanatory variable is expressed as follows:

$$\beta_{0\,j}=\gamma_{00}+\gamma_{01}\,W_{j}+u_{0\,j}$$

where γ_00_ is the level-2 intercept, γ_01_ is the regression coefficient of the neighborhood-level explanatory variable *W*_*j*_ (i.e., the average value of trust for each neighborhood), and *u*_0__*j*_ is the level-2 residuals (i.e., level-2 random effect). That is, these models predict the individual-level dependent variable using individual-level independent variables (i.e., individual-level trust and covariates) and the neighborhood-level independent variable (i.e., neighborhood-level trust). A statistically significant γ_01_ means that neighborhood-level trust explains the variation in the dependent variable among neighborhoods, in other words, that neighborhood-level trust is associated with the dependent variable. Since this study used two dependent variables (perceived neighbors’ cooperative behaviors and respondents’ own cooperative behaviors), we conducted two multilevel model analyses applied to each.

Guided by Kreft and de Leeuw (1998), in order to avoid multicollinearity between individual- and neighborhood-level trust, they were centered on group mean and grand mean, respectively. That is, for neighborhood-level trust, we subtracted the grand mean of trust (2.464) from each neighborhood’s average value of trust. For individual-level trust, the average value of neighborhood (i.e., group mean) in which each individual was embedded was subtracted from each individual’s value of trust. The number of neighborhood-level observations was 26, and that of individual-level was 476, with an average of 18.3 respondents per neighborhood (standard deviation was 7.5).

We used *spmat*, *spreg*, and *mixed* commands of Stata 15.1 (Stata Corp, Texas, United States) for creating the inverse-distance spatial-weighting matrix, running the spatial Durbin model analyses, and running the multilevel model analyses, respectively.

## Results

Table 2 shows the descriptive statistics of the participants. The number of men and women were almost the same, and those with educational background beyond university graduate were about 30%. Based on the responses on the four-point Likert scales, means of respondents’ cooperative behaviors and their perceived neighborhood residents’ cooperative behaviors were 1.71 and 2.98, respectively.

**TABLE 2:** Descriptive statistics (*n* = 476).

	***n***	**%**
**Sex**		
Male	243	51.1
Female	233	49.0
**Educational level**		
College/vocational school or less	323	67.9
University degree or higher	153	32.1
**Perceived social class**		
Lowest	83	17.4
Lower middle	207	43.5
Upper middle	111	23.3
Highest	75	15.8
**Presence of elementary school children**		
Presence	41	8.6
Absence	435	91.4

	**Mean**	**SD**

Age	50.24	12.34
Residence year	32.86	19.81
Trust (respondent’s own value) (1–4)	2.46	0.67
Fear of crime (1–4)	2.58	0.72
Perceived neighbors’ cooperative behavior (1–4)	2.98	0.73
Respondents’ cooperative behavior (1–4)	1.71	0.57

Table 3 shows results of the spatial Durbin models. Perceived neighbors’ cooperative behaviors was positively associated with subjective social class, residence year, the presence of elementary school children in the household, and fear of crime. Respondents’ trust, as well as neighborhood trust weighted by the inverse distance (spatial lag term), also had statistically significant positive associations with perceived neighbors’ cooperative behaviors. In addition, the dependent variable showed statistically significant spatial autocorrelation (rho). This meant that perceived neighborhood residents’ cooperation was spatially dependent on each other.

**TABLE 3:** Spatial Durbin model estimates for perceived neighbors’/respondents’ cooperative behaviors (*n* = 476).

	**Perceived neighbors’ cooperative behaviors**		**Respondents’ cooperative behaviors**	
	**Coefficients**	**95% CI^a^**	**Coefficients**	**95% CI^a^**
Sex				
Male	–0.092	(−0.230, 0.046)	0.047	(−0.053, 0.147)
Female	Reference		Reference	
Age	0.006	(−0.001, 0.012)	0.018^∗^	(0.014, 0.022)
Education level				
College/vocational school or less	Reference		Reference	
University degree or higher	0.105	(−0.042, 0.252)	0.117^∗^	(0.012, 0.222)
Perceived social class				
Lowest	Reference		Reference	
Lower middle	0.108	(−0.073, 0.288)	0.001	(−0.129, 0.132)
Upper middle	0.106	(−0.098, 0.310)	–0.017	(−0.163, 0.128)
Highest	0.278^∗^	(0.051, 0.505)	–0.039	(−0.203, 0.124)
Residence year	0.004^∗^	(0.001, 0.008)	0.003	(0.000, 0.006)
Presence of children				
Presence	0.424^∗^	(0.190, 0.659)	0.295^∗^	(0.124, 0.465)
Absence	Reference		Reference	
Fear of crime	0.066^∗^	(0.000, 0.132)	0.122^∗^	(0.075, 0.170)
Trust	0.151^∗^	(0.058, 0.245)	0.131^∗^	(0.063, 0.199)
Trust (Spatial lag)	2.327^∗^	(1.376, 3.278)	0.174^∗^	(0.035, 0.313)

Rho	1.327^∗^	(1.296, 1.359)	–0.506	(−1.960, 0.947)

*^*a*^CI = confidence interval; ^∗^*p* < 0.05.*

Next, respondents’ cooperative behaviors was associated with age, educational level, the presence of elementary school children in the household, and fear of crime. Individual trust, as well as spatially weighted neighborhood trust, were positively related to the respondents’ cooperative behaviors. On the other hand, respondents’ cooperative behaviors did not have a statistically significant spatial autocorrelation.

Table 4 shows the results of the multilevel models. Perceived neighborhood cooperative behaviors was associated with residence year, the presence of elementary school children in the household, and fear of crime. Individual- and neighborhood-level trust were not statistically significant. In addition, the variance component was small, indicating that there is a slight unexplained variance in the perceived neighbors’ cooperation among neighborhoods.

**TABLE 4:** Multilevel estimates for perceived neighbors’/respondents’ cooperative behaviors (individual-level *n* = 476, neighborhood-level *n* = 26).

	**Perceived neighbors’ cooperative behaviors**		**Respondents’ cooperative behaviors**	
	**Coefficients**	**95% CI^a^**	**Coefficients**	**95% CI^a^**
*Individual-level variables*				
Intercept	2.715^∗^	(2.382, 3.048)	0.916^∗^	(0.675, 1.157)
Sex				
Male	–0.062	(−0.196, 0.072)	0.039	(−0.059, 0.137)
Female	Reference		Reference	
Age	0.000	(−0.006, 0.007)	0.013^∗^	(0.009, 0.018)
Education attainment				
College/vocational school Or less	Reference		Reference	
University degree or higher	0.027	(−0.119, 0.174)	0.072	(−0.035, 0.178)
Perceived social class				
Lowest	Reference		Reference	
Lower middle	0.030	(−0.151, 0.212)	–0.061	(−0.193, 0.071)
Upper middle	–0.022	(−0.228, 0.183)	–0.099	(−0.248, 0.051)
Highest	0.215	(−0.010, 0.441)	–0.093	(−0.257, 0.071)
Residence year	0.006^∗^	(0.002, 0.010)	0.004^∗^	(0.001, 0.007)
Presence of children				
Presence	0.387^∗^	(0.157, 0.618)	0.255^∗^	(0.087, 0.422)
Absence	Reference		Reference	
Fear of crime	0.071^∗^	(0.007, 0.136)	0.121^∗^	(0.074, 0.168)
Trust	0.076	(−0.022, 0.174)	0.073^∗^	(0.001, 0.144)
*Neighborhood-level variable*				
Trust	0.119	(−0.352, 0.591)	0.178	(−0.136, 0.492)

Variance component (intercept)	0.005	(0.000, 0.138)	0.000	(0.000, 0.000)

*^*a*^CI = confidence interval; ^∗^ p < 0.05.*

Respondents’ cooperative behaviors was positively associated with age, residence year, the presence of elementary school children, and fear of crime. While individual-level trust had a statistically significant positive association with respondents’ cooperative behaviors, neighborhood-level trust was not statistically significantly related to it. Similar to the model of perceived neighbors’ cooperation, there is a slight unexplained variance among neighborhoods.

## Discussion

The spatial Durbin model analyses showed that neighborhood trust had positive associations with perceived neighbors’/respondents’ cooperative behaviors. The important point of this study is that when we examined the relationships between the neighborhood-level (*choumoku*) trust and individual-level cooperative behaviors using the conventional multilevel models, the relationships between them were not statistically significant. One reason for these contrasting results between spatial models and multilevel models may be that the variances in the dependent variables among neighborhoods were very small in multilevel models. The neighborhood effects of trust addressed in this study may have occurred with neighborhood residents living very nearby, and the analyses using administrative districts might not be appropriate for capturing it. If we had analyzed our data using only conventional multilevel models with a single unit of the neighborhood, our study conclusion would have been that “neighborhood trust was not associated with cooperative behaviors.”

The implication of this study is that the influence from neighbors who live closer may be more important than that of a neighborhood unit such as a *choumoku*. That is, while the effect of trust seems to have a spatial spillover effect, our analyses suggest that this may not be captured by using neighborhood geographical districts such as a multilevel model that ignores the proximity between the respondents. As mentioned in the introduction, while it is fairly obvious that cooperative behaviors occur when surrounded by neighbors who trust each other (e.g., Ahn and Ostrom, 2008; Yamagishi, 2011), the neighbors mentioned here are not those who merely live within the same geographical district but those who live in the closer neighborhood (specifically, in terms of geographical range where influence actually reaches). The reason for this is obvious: while trust among neighborhood residents–who are likely to have social interactions–is important for people’s cognition (e.g., intention to behave), the importance of ‘distant neighbors’ who are unlikely to have actual social interactions is low. The multilevel model analyses included information on residents who had a weak neighborhood effect as “noise” in neighborhood-level variables.

However, in a society wherein people’s social influence processes are clearly defined by a geographical boundary, results different to this study may be found. For example, in areas where administrative neighborhood units such as a school district undertake social activities (e.g., neighborhood watch), analyses using the units (e.g., a multilevel model) may well explain the variance of the dependent variable.

Some limitations of this study should be noted. First, for variables used in the present study, reliability and validity were not established. For example, perceived neighbors’ cooperative behaviors contained only two items, indicating low Cronbach’s alpha value (0.613), and was mainly relevant to neighborhood watch activities, suggesting low validity as items to measure the concept of cooperative behaviors. In addition, trust, the independent variable of this study, was measured by only one item. Although this item was extracted from the General Trust Scale (Yamagishi and Yamagishi, 1994), whose reliability and validity have been repeatedly confirmed (Yamagishi, 1988; Yamagishi and Cook, 1993; Yamagishi et al., 2005, 2015), and was one of the most representative items of the scale, using only a single item did not guarantee reliability and validity. Future studies need to examine whether the spatial analysis proposed in this study is effective in psychological research, using a set of psychological scales whose reliability and validity are established.

Second, this study used 4-point Likert-type scales for measuring the dependent and independent variables because Japanese respondents are more likely to choose the midpoint when odd-numbered options are used (Chen et al., 1995). However, the small number of options may contribute to the low reliability coefficients for the variables of this study (Churchill and Peter, 1984; Weng, 2004). Third, although residents of urban areas of Japan living in a large apartment building are likely to have social interactions mainly within the building, this study used data from only one respondent from each apartment building. This may have led to an underestimation of the neighborhood effect. Fourth, the null findings of the effects of neighborhood-level trust in multilevel models may be attributable to the small number of neighborhood-level observations (*n* = 26). In addition, the geographical size of neighborhood units used in the multilevel models (*choumoku*) might be too small. Fifth, since we conducted this study in one of Japan’s metropolitan areas, the generalizability of our findings is limited.

Nevertheless, this study suggested that the spatial model may be appropriate for modeling a certain type of neighborhood effect in society more precisely. On the other hand, multilevel models or ecological analyses that use a more understandable unit of analysis may be more convenient when communicating with policy makers/practitioners and developing neighborhood intervention methods. It is essential to choose an appropriate approach with theoretical and practical considerations to understand the relationships between independent and dependent variables examined.

## Data Availability Statement

The datasets generated for this study are available on request to the corresponding author.

## Ethics Statement

The studies involving human participants were reviewed and approved by the Ethics Committee at Graduate School of Humanities and Sociology, The University of Tokyo. Written informed consent for participation was not required for this study in accordance with the national legislation and the institutional requirements.

## Author Contributions

DT contributed to developing the theoretical framework, data collection, data analysis, and overall writing. TS contributed to data analysis and revising the manuscript critically.

## Conflict of Interest

The authors declare that the research was conducted in the absence of any commercial or financial relationships that could be construed as a potential conflict of interest.
